# Turner syndrome across the lifespan: a 25-year single-center experience from neonatal diagnosis to adult outcomes

**DOI:** 10.3389/fendo.2026.1771065

**Published:** 2026-04-10

**Authors:** Aysegul Ceran, Sirmen Kızılcan Çetin, Zeynep Şıklar, Elif Özsu, Merih Berberoğlu, Zehra Aycan

**Affiliations:** Department of Pediatric Endocrinology, Ankara University Faculty of Medicine, Ankara, Türkiye

**Keywords:** final height, growth hormone treatment, multisystem comorbidities, transition to adult care, turner syndrome

## Abstract

**Background:**

Turner syndrome (TS) is a lifelong chromosomal disorder characterized by short stature, gonadal dysgenesis, and multisystem comorbidities. This study presents a 25-year singlecenter experience, evaluating growth outcomes—including the impact of growth hormone (GH) initiation age—pubertal development, comorbidity burden, and transition patterns from pediatric to adult care.

**Methods:**

This retrospective cohort study included 31 TS patients followed between 1996 and 2021. Clinical, anthropometric, and laboratory data at diagnosis, during follow-up, and at the most recent visit were collected using structured forms. Karyotype, GH treatment history, final height, pubertal status, systemic comorbidities, and adult follow-up outcomes were analyzed. Adult TS patients were re-evaluated using a standardized adult TS assessment form. Final height outcomes were compared across GH initiation age groups (≤6 y, 6–12 y, ≥12 y). Continuous variables were assessed for normality and compared using ANOVA or Kruskal–Wallis tests, as appropriate. Multivariable linear regression analysis was performed to evaluate independent predictors of final height standard deviation score (SDS). Categorical variables were summarized descriptively.

**Results:**

The cohort included 31 patients (current age range: 1.5–38 years); 32% had classical 45, X and 68% mosaic karyotypes. Twenty-three patients received GH therapy, with a mean initiation age of 7.4 ± 4.1 years. Mean final height was 155.7 ± 6.8 cm. Patients who initiated GH therapy at ≤6 years achieved higher final heights (approximately 159–163 cm) compared with those initiating at ≥12 years (approximately 140–156 cm). In multivariable analysis, earlier GH initiation showed a trend toward association with improved final height SDS. Multisystem comorbidities were frequent and, in descending order of prevalence, included renal anomalies (41%), cardiovascular abnormalities (35%), hearing impairment (34%), ophthalmologic disorders (32%), autoimmune disease (29%), and metabolic disorders (25%). Among adult patients who transitioned to adult care (n = 13), only one maintained regular endocrinology follow-up, and none completed recommended aortic surveillance.

**Conclusions:**

Early GH initiation yields final height outcomes comparable to the most favorable international cohorts. Turner syndrome requires lifelong, multidisciplinary follow-up, yet major gaps persist during transition to adult care. Strengthening structured transition pathways is essential to optimize long-term outcomes.

## Highlights

What’s known:

- Early GH therapy improves growth outcomes in Turner syndrome.
- TS requires lifelong surveillance due to multisystem comorbidities.

What is new:

- Early GH initiation (≤6 y) produced a ~7 cm final height advantage over late initiation.
- Real-world long-term outcomes remain substantially below clinical trial achievements.

## 1 Introduction

Turner syndrome (TS) is a chromosomal disorder resulting from partial or complete monosomy X and affects approximately 1 in 2, 500 live female births. Its clinical spectrum is highly heterogeneous, encompassing short stature, gonadal dysgenesis, congenital cardiovascular and renal anomalies, autoimmune disorders, metabolic complications, and neurocognitive vulnerabilities. Since these manifestations evolve across childhood, adolescence, and adulthood, TS is recognized as a lifelong condition requiring coordinated multidisciplinary care. Early diagnosis and systematic surveillance are central to preventing morbidity and improving long-term health outcomes (1, 2).

Short stature remains one of the earliest and most consistent features of TS, and growth hormone (GH) therapy is the cornerstone of height optimization. Multiple clinical trials have demonstrated that early GH initiation—ideally before 6 years of age—leads to superior final Height, particularly when combined with individualized estrogen replacement during adolescence. However, real-world outcomes vary widely due to delayed diagnosis, inconsistent treatment duration, heterogeneity in estrogen induction timing, and disparities in healthcare access. Comparing real-life cohorts with structured trial-based evidence remains essential for understanding these gaps and guiding clinical decision-making (2–5).

Beyond growth outcomes, TS is associated with significant lifelong comorbidities, including structural heart disease, aortic dilation, hypertension, renal malformations, thyroid autoimmunity, hearing loss, and metabolic disease. Despite international guideline recommendations for ongoing cardiovascular imaging, annual endocrine follow-up, and comprehensive adult transition, adherence to these standards is frequently suboptimal. The transition from pediatric to adult care is particularly vulnerable, and poor transition contributes to late detection of high-risk complications and preventable morbidity (1, 2, 6, 7).

This study presents a 25-year single-center experience, evaluating growth outcomes—including the impact of GH initiation age, multisystem comorbidities, pubertal development, and transition success in TS. By integrating updated final height data with detailed long-term comorbidity profiles, this cohort provides real-world evidence to contextualize current guideline recommendations and highlight persistent gaps in lifelong TS management.

## 2 Methods

This retrospective descriptive study included patients diagnosed with TS who were regularly followed at our Pediatric Endocrinology Clinic, between 1996 and 2021. All participants had confirmed karyotype analyses. Standard diagnosis was established using at least 30 metaphase spreads obtained from peripheral lymphocytes.

A structured data collection form was used to collect anthropometric, clinical, and laboratory data. Datas included: age at diagnosis, karyotype, anthropometric and physical examination findings at diagnosis, during follow-up, and at the most recent visit; history of GH therapy, including treatment duration, side effects, and response to therapy; presence or absence of spontaneous puberty; initiation of hormone replacement therapy (HRT) for pubertal induction; and findings related to ophthalmologic, otologic, renal, cardiovascular, autoimmune, gastrointestinal, metabolic, and psychiatric/systemic comorbidities. Final height outcomes were analyzed across GH initiation age groups (≤6 y, 6–12 y, ≥12 y).

Growth hormone (GH) therapy was initiated and managed in accordance with contemporary international TS guidelines (2). GH treatment was started at an initial dose of approximately 50 µg/kg/day, administered as daily subcutaneous injections. The GH dose was not kept strictly constant throughout follow-up; instead, dose adjustments were performed periodically based on body weight changes, growth response, and clinical tolerance, in line with routine clinical practice (2). Patients on GH therapy were monitored at regular intervals (every 3–6 months) using a standardized follow-up protocol.

GH dose adjustments were considered in cases of: suboptimal growth velocity despite reported adherence, significant weight gain requiring dose recalculation, elevated IGF-1 levels or emerging metabolic abnormalities, development of treatment-related adverse effects.

GH therapy was discontinued according to guideline-based criteria, including: marked decline in growth velocity (<2 cm/year), advanced bone age with near-complete epiphyseal closure, achievement of near-final height, occurrence of significant adverse events necessitating treatment cessation (e.g. development of type 2 diabetes mellitus).

For patients who had transitioned to adult endocrinology care (aged ≥18 years), follow-up information was collected. After obtaining informed consent, patients were invited to the clinic and evaluated using an ‘Adult Data Collection Form’. This form assessed whether patients had received regular multidisciplinary follow-up for TS and appropriate systemic screenings, any newly identified adult-onset comorbidities, ongoing treatments, and their current academic and social status.

The study protocol was approved by the Local Ethics Committee (Approval No: [I08-504-22], Date: [15.09.2022]). Written informed consent was obtained from all adult participants, and for children and adolescents, patients’ and parental consent were obtained in accordance with institutional guidelines and the Declaration of Helsinki. Continuous variables were tested for normality using the Shapiro–Wilk test and are presented as mean ± SD or median, as appropriate. Categorical variables are expressed as counts and percentages. Final height SDS was compared across GH initiation age groups (≤6 years, 6–12 years, ≥12 years) using one-way ANOVA or Kruskal–Wallis test with *post-hoc* pairwise comparisons (Bonferroni-adjusted). To evaluate independent predictors of final height outcomes, multivariable linear regression analysis was performed with final height SDS as the dependent variable. The following clinically relevant covariates were included in the model: age at GH initiation, duration of GH therapy (years), baseline height SDS). Regression coefficients are reported as β estimates with 95% confidence intervals (CI). A two-sided p value < 0.05 was considered statistically significant. All statistical calculations were performed using Statistical Package for the Social Sciences for Windows, version 22.0 (IBM Inc., Armonk, NY, USA).

## 3 Results

A total of 31 patients with TS who were regularly followed at our center were included in the study (**Table 1**). The current ages ranged from 1.5 to 38 years. The age at diagnosis ranged widely from the antenatal period (n=4, 13%) and neonatal period (n=5, 16%) to 16.5 years, with a mean age at diagnosis of 8.0 ± 5.5 years. At the time of data collection, 15 patients (49%) were aged 18 years or older. Karyotype analysis revealed classical 45, X in 10 patients (32%) and mosaicism in 21 patients (68%); the most common mosaic karyotype was 45, X/46, XX, observed in 9 patients (29%). Three patients (10%) had a ring X chromosome, and two patients (6%) had an isochromosome Xq (i[Xq]) (**Table 2**).

**Table 1:** Growth hormone treatment characteristics and final height data in turner syndrome (n=23).

Case no	GH start age (y)	Baseline height SDS	GH duration (yrs)	Final height (cm)	Final height SDS	Genetic target SDS deviation
2	2.21	-2.18	12	161	-0.35	-0.63
3	6.5	-2.07	8	151.5	-1.47	-0.78
4	5.3	-1.29	7.74	152.5	-1.87	-1.58
5	4.8	-1.66	9.18	158	-0.87	-0.04
6	5.6	-4.68	10.67	151	-2.06	-1.42
7	12	-2.19	2.92	152	-1.89	-0.24
8	13	-2.5	3	158.5	-0.71	-0.91
9	9	-1.57	7	148.8	-1.93	-0.82
10	11.6	-4.73	4	147.4	-2.67	-1.78
12	3	-1.58	8.34	160.5	-0.44	-0.79
15	5.5	-2.48	4.35	157.5	-0.95	-0.52
16	12.7	-3.93	0.97	156	-1.21	-0.3
17	3.9	-3.22	8.7	148.7	-2.45	-1.52
18	6.9	-2.88	6.51	148	-2.5	-0.04
19	3.6	-3.61	13.75	152	-1.89	-1.31
20	11.8	-2.71	2.5	156.3	-1.16	1.04
22	11.5	-3.14	3.39	151.5	-1.98	0.32
23	15	-4.07	3	143	-3.42	-3.29
24	5.1	-2.2	7.2	162.5	-0.1	-2.43
25	10.1	-2.18	2.5	161	-0.36	-1.43
27	14.8	-5.9	3.67	146	-2.93	-0.99
28	7.2	-3.24	9.6	154	-1.55	-1.25
31	15.7	-4.31	0.375	140	-3.93	-2.68

**Table 2:** Chromosomal karyotypes and frequency of systemic ınvolvement in turner syndrome.

Pathology	45, X (n)	Mosaic (n)	Structural (n)	Isochromosome (n)
Bicuspid Aortic Valve	10	7	3	3
Coarctation of Aorta	2	0	0	0
Horseshoe Kidney	8	7	3	3
Rotational Abnormalities	2	0	0	0
Hypothyroidism	4	3	7	7
Positive Anti-TPO	2	3	3	3
Positive Anti-Tg	2	3	3	3
Recurrent top Ear Infections	8	7	3	3
Conduction Hearing Loss	4	3	3	3
Sensorineural Hearing Loss	0	2	0	0
Osteopenia/Osteoporosis	6	7	10	7
Scoliosis	3	2	2	2
Obesity	6	10	7	7
Overweight	4	3	3	3
Insulin Resistance	3	2	0	0

Short stature was the most common presenting feature, observed in 15 patients (48%). Combined short stature and delayed puberty were present in 3 patients (9.6%). Other initial presentations included lymphedema in 5 patients (16%), antenatal diagnosis in 4 patients (12.9%), mental–motor retardation in 2 patients (6.4%), oligomenorrhea in 1 patient (3.2%), and goiter in 1 patient (3.2%). Younger children aged 2–7 years were more frequently diagnosed antenatally in recent years, likely reflecting improved prenatal screening practices.

At initial evaluation, the mean height SDS was −2.3 ± 1.6 (range, −5.9 to 0.8), and BMI percentiles ranged from 72 to 139. Five patients (n=5, 16%) met the criteria for obesity. Growth hormone (GH) therapy was not initiated in 8 patients (n=8, 26%) for the following reasons: lack of deviation from the growth curve in 2 patients (n=2, 6%), absence of family consent in 1 patient (n=1, 3%), severe motor–cognitive delay with spasticity in 1 patient (n=1, 3%), and presentation at >18 years of age in 4 patients (n=4, 13%).

Twenty-three patients received GH therapy. Based on the updated dataset, the mean age at GH initiation was 7.4 ± 4.1 years (range: 2.2–15.7). The mean pre-treatment Height SDS was −2.82 ± 1.19 (range: −5.90 to −1.29). The initiation dosage was 50 µg/kg/day.

Adverse effects observed during therapy included glucose intolerance in 3 patients (n=3, 9.6%), type 2 diabetes mellitus (T2DM) in 1 patient (n=1, 3.2%), elevated alanine aminotransferase (ALT) levels in 2 patients (n=2, 6.4%), a psychotic episode in 1 patient (n=1, 3.2%), and progression of scoliosis in 3 patients (n=3, 9.6%). Growth hormone (GH) treatment was discontinued at 12.5 years of age in the patient who developed T2DM. The two patients with elevated ALT levels were evaluated by hepatology; one was diagnosed with hepatitis B infection and the other with idiopathic chronic liver disease, and GH therapy was continued in both patients under close monitoring.

The mean GH treatment duration in the updated dataset was 6.3 ± 3.7 years (range: 0.38–13.75 years). GH therapy was managed with periodic dose adjustments and systematic monitoring. Discontinuation was primarily driven by reduced growth velocity and advanced bone age, consistent with current Turner syndrome guidelines (2).

Final height data were available for all 23 GH-treated patients. The mean final Height was 155.7 ± 6.8 cm (range: 140–162.5 cm), and the mean final Height SDS was −1.37 ± 1.02 (range: −3.93 to 1.04). Final height SDS tended to be higher in patients who initiated GH therapy at an earlier age (**Supplementary Table S1**; however, differences across GH initiation age groups did not reach statistical significance (p = 0.09). In multivariable linear regression analysis including GH initiation age, GH treatment duration, and baseline height SDS, no variable remained independently associated with final height SDS. Earlier GH initiation and higher baseline height SDS showed trend-level associations with higher final height SDS, but these did not reach conventional statistical significance. The model explained approximately 42% of the variance in final height SDS (adjusted R² = 0.42).

Overall, 43% of patients achieved a final height above −2 SDS. The mean deviation from genetic target height was −0.94 ± 1.01 SDS, indicating that most patients approached—but did not fully reach—their genetic height potential.

Patients who initiated GH therapy at or before 6 years of age (n=9) demonstrated markedly superior growth outcomes compared with those who initiated treatment later. Their final heights ranged between approximately 159 and 163 cm, with a mean final height SDS of −0.66 ± 0.36. In contrast, patients who started GH therapy at 12 years or older (n=7) reached substantially lower final heights, ranging from 140 to 156 cm, and their mean final Height SDS was −2.27 ± 1.15. These findings indicate that early initiation of GH therapy is associated with an approximate 9–10 cm advantage in final Height.

Among the eight patients who were not on GH therapy, none achieved a final height above −2 SDS. Adult heights ranged from 138 to 150 cm, and all untreated patients had final height SDS values below −2.5, further emphasizing the clear benefit of GH treatment in optimizing height outcomes. When comparing pediatric and adult final height data among GH-treated patients, the pediatric subgroup (n = 5) had a mean height of 148.9 ± 6.7 cm, whereas the adult subgroup (n = 18) achieved a higher mean final height of 157.8 ± 5.9 cm. This difference likely reflects earlier GH initiation and longer total treatment duration in the adult subgroup.

### 3.1 Pubertal development

Pubertal induction was required in 11 patients (n=11, 35.5%) and was initiated at a mean age of 13.8 ± 1.4 years. Spontaneous puberty occurred in 12 patients (n=12, 38.7%), predominantly among those with mosaic karyotypes; however, a substantial proportion showed arrested pubertal progression and subsequently required hormone replacement therapy (HRT). Estrogen replacement was initiated in 19 patients (n=19, 61.3%) using either transdermal 17β-estradiol (n=5, 16.1%) or oral 17β-estradiol (n=14, 45.2%), starting at a low dose with stepwise escalation every 6 months and reaching adult replacement doses over approximately 2–3 years; progestin was typically added after 12–24 months of estrogen therapy or upon the occurrence of breakthrough bleeding. In late adolescence/adulthood, only 4 patients (n=4, 12.9%) maintained regular spontaneous menses, while the remaining patients required ongoing estrogen–progestin replacement due to ovarian insufficiency; among these, three had 45, X/46, XX mosaicism (n=3, 9.6%) and one had 45, X/46, Xr(X) (n=1, 3.2%).

### 3.2 Systemic comorbidities

Cardiovascular anomalies were identified in 11 patients (n=11, 35%), including bicuspid aortic valve, persistent left superior vena cava, complex congenital heart defects, aberrant ventricular bands, and mild valvular insufficiencies. Hypertension was detected in 3 patients (n=3, 9.6%). Renal anomalies were present in 13 patients (n=13, 41%), most commonly horseshoe kidney and pelvicalyceal dilatation. Conductive hearing loss was identified in 7 patients (n=7, 22.6%), typically secondary to recurrent otitis media, and ophthalmologic abnormalities, including refractive errors and strabismus, were also observed in 7 patients (n=7, 22.6%). Autoimmune disease occurred in 9 patients (n=9, 29%), predominantly autoimmune thyroiditis. Gastrointestinal findings included hepatitis B infection (n=1, 3.2%) and idiopathic chronic liver disease (n=1, 3.2%). Dermatologic manifestations included multiple nevi, vitiligo, alopecia, psoriasis, and lymphedema. Scoliosis was documented in 6 patients (n=6, 19.4%), while osteoporosis or osteopenia was identified in 3 of the 5 patients evaluated by dual-energy X-ray absorptiometry (DEXA). Metabolic comorbidities included obesity in 8 patients (n=8, 25%), impaired glucose tolerance, type 2 diabetes mellitus (T2DM), and nonalcoholic fatty liver disease (NAFLD). Neuropsychiatric diagnoses included depression, anxiety, obsessive–compulsive disorder (OCD), and intellectual disability.

### 3.3 Adult follow-up

Of 13 patients transferred to adult care, only one continued regular endocrinology follow-up. None had undergone recommended aortic aneurysm surveillance.

The mean age of adult patients was 27.4 ± 5.1 years (range: 21–38).

Updated final/adult Height in this subgroup was 157.8 ± 5.9 cm, with final heights ranging from 140 to 162.5 cm.

Adult-onset comorbidities included ankylosing spondylitis, epilepsy, pericardial effusion, splenic strangulation, major depressive disorder, hypertension, and osteoporosis. One patient had persistent liver enzyme elevation attributed to TS.

### 3.4 Academic and reproductive outcomes

Among adult patients with Turner syndrome, 3 patients (n=3, 33%) had completed a university or associate degree, 2 patients (n=2, 22%) were currently enrolled in university, 3 patients (n=3, 33%) were attending open high school programs, and 1 patient (n=1, 11%) was pursuing a PhD. Five patients (n=5, 56%) were married at the time of last follow-up. Assisted reproductive techniques had been used by 2 patients (n=2, 22%), one of whom experienced two miscarriages. One patient (n=1, 11%) with a complex mosaic karyotype (45, X [95%]/46, XX [2%]/47, XXX [3%]) achieved pregnancy at 30 years of age and delivered a healthy infant by cesarean section at 38 weeks of gestation due to cephalopelvic disproportion. No major maternal or neonatal complications were observed during the peripartum period. No spontaneous or assisted pregnancies were documented in the remaining adult patients during follow-up.

## 4 Discussion

This 25-year single-center study provides a comprehensive real-life overview of growth outcomes, pubertal development, multisystem involvement, and transition patterns in patients with TS. Our cohort reveals several significant convergences and divergences that highlight both advances and persistent clinical gaps in TS management.

In our cohort, short stature was the most common presenting feature, consistent with previous reports (2, 8–10). Short stature affects nearly 95% of individuals with TS, with untreated adult height approximately 20 cm below population and target height averages (10). Growth deceleration typically begins in early childhood and progresses with age. This impairment is largely attributed to haploinsufficiency of genes located in the pseudoautosomal regions (PAR1 and PAR2) of the X chromosome, which escape X-inactivation. Among these, *SHOX* plays a pivotal role in growth plate regulation and longitudinal bone growth, and reduced gene dosage results in characteristic skeletal disproportions and diminished growth potential (2, 8–10). Mosaic karyotypes, partial preservation of X-linked gene expression, are generally associated with milder growth and pubertal phenotypes (2). In our cohort, the differences between classical 45, X and mosaic patients were no longer evident after adjustment for GH therapy timing and duration, indicating that treatment-related factors may be more influential than karyotype alone. Untreated TS patients reached an average adult height of approximately 141 cm (11, 12), while GH therapy has been shown to improve final height by around 6.5 cm, typically achieving 147–150 cm (2, 13). Across large cohorts, GH therapy in TS consistently produces meaningful but variable improvements in growth. In the literature, the international series (14–16) collectively show average gains of ~3 cm relative to predicted final height, with substantial interindividual variation—ranging from −4.7 cm to +12.1 cm—and 25% of patients achieving ≥5 cm improvement (15). In contrast to randomized or registry-based studies, the association between early GH initiation and final height in our real-world cohort did not reach statistical significance after multivariable adjustment. This likely reflects the limited sample size, heterogeneity in treatment duration, and delayed diagnosis in some patients. Nevertheless, the consistent trend toward better height outcomes with earlier GH initiation is compatible with existing literature and supports early diagnosis and timely treatment as clinically relevant goals. GH-treated patients in our study achieved a mean final height of 155.7 ± 6.8 cm, corresponding to a mean ΔHSDS of approximately +1.0 SDS, comparable to the +1.17 ΔHSDS reported in prepubertal patients after 3 years (14). Notably, the magnitude of improvement in our early starters (≤6 years)—a final height SDS of −0.66 and attained heights of 159–163 cm—mirrors the most favorable outcomes seen in the Backeljauw et al.’s cohort (14), reinforcing that early GH initiation is the single strongest driver of height potential. In contrast, our late initiators (≥12 years) reached substantially lower adult heights (140–156 cm; final Height SDS −2.27), consistent with the diminished growth response observed in older or pretreated groups (8, 9), which showed only a +0.1 ΔHSDS in pretreated patients. The untreated patients in our study—none of whom achieved a final height above −2 SDS—also reflect the natural history observed prior to widespread GH use, similar to the non-GH-treated patients described (2, 4, 14, 16). These results highlight the importance of early diagnosis, individualized dosing, and careful longitudinal follow-up, while also underscoring that some patients may have limited response due to constitutional factors or comorbidities.

The majority of patients in our cohort were diagnosed relatively late. Although the increasing use of prenatal ultrasonography and genetic testing has improved early detection (2), many cases—particularly those with subtle clinical findings or mosaic karyotypes—remain undiagnosed until school age or adolescence, delaying timely interventions. Early diagnosis is essential not only for initiating GH therapy and pubertal induction but also for implementing early cardiovascular, renal, and systemic screening to reduce long-term morbidity and mortality. In our series, three patients were identified prenatally due to significant TS stigmata, of whom two had mosaic karyotypes (45, X/46, XX) and one had a non-mosaic 45, X karyotype, underscoring the variability of phenotype regardless of chromosomal pattern. The subsequent termination of 45, X pregnancies may contribute to the higher proportion of mosaic cases among survivors. These findings emphasize the need for comprehensive, individualized follow-up from diagnosis through adulthood, regardless of the mode or timing of diagnosis.

Pubertal development remains a major concern in TS. Spontaneous puberty occurred in 12 patients, predominantly among mosaic karyotypes (especially 45, X/46, XX), consistent with international reports showing that 20–40% of mosaic TS patients exhibit spontaneous thelarche and menses. The fact that only four patients maintained regular cycles into adulthood (2). Nonetheless, the majority required estrogen replacement, emphasizing the need for timely initiation of individualized pubertal induction regimens. While spontaneous pregnancies occur in 4.8–6.7% of TS patients, these are often accompanied by high miscarriage rates (up to 45%) and fetal malformations (up to 34%) (2). A study including 22 mosaic TS patients (17 with 45, X/46, XX and 5 with 45, X/46, XX/47, XXX) undergoing IVF reported a pregnancy rate of 8.6% and a live birth rate of 5.7%, with no significant difference between mosaic subtypes (17). Only a minority of mosaic TS patients conceive within the first two years of marriage, and neither age at menarche nor age at marriage appears to influence fertility outcomes. In our series of 31 patients, one mosaic case achieved pregnancy at age 30 via IVF and delivered a healthy infant. This underscores the importance of appropriate transition counseling and monitoring, particularly for mosaic TS patients, to preserve fertility potential.

In our cohort, the prevalence of cardiovascular (35%), renal (41%), autoimmune (29%), metabolic (25%), and auditory complications closely aligns with real-world data from national and international cohorts across Europe and other regions (2). Large observational studies report congenital heart defects in up to 50% of patients, with bicuspid aortic valve (~30%) and aortic coarctation (~15%) being the most frequent findings, alongside acquired complications such as hypertension, aortic dilation, and increased cerebrovascular risk in adulthood (2, 10). The cardiovascular profile observed in our cohort therefore reflects typical real-world disease expression rather than center-specific bias, although incomplete adherence to TS-specific surveillance—particularly in earlier decades—may have led to underdiagnosis of progressive aortic pathology.

Renal anomalies were identified in approximately one-third of patients in our cohort, consistent with real-world reports describing horseshoe kidney, malrotation, ectopic kidney, duplex systems, and renal agenesis in 30–40% of individuals with TS (10). Although many renal anomalies are clinically silent, their association with hypertension and recurrent urinary tract infections underscores the need for systematic screening and long-term follow-up. Similarly, the high frequency of hearing complications observed in our patients mirrors population-based data indicating that more than 50% of women with TS develop hearing loss between 15 and 35 years of age, with progressive worsening over time (10).

Endocrine-metabolic comorbidities, including autoimmune thyroiditis, glucose intolerance, type 2 diabetes mellitus, obesity, and hepatic abnormalities, were also common in our cohort and comparable to real-world prevalence estimates. Liver enzyme elevations and chronic liver disease, which increase with age and may affect up to 30–50% of adults with TS, are well-recognized non-autoimmune manifestations related to metabolic, vascular, and biliary involvement rather than treatment effects alone (10). Importantly, while GH therapy may transiently induce insulin resistance, existing real-world evidence indicates that this effect is generally reversible after treatment cessation, supporting the metabolic safety of GH when appropriately monitored (2, 10). Adverse events potentially related to GH therapy were infrequent, clinically monitored, and generally reversible. No cardiovascular comorbidity was temporally or clinically attributed to GH therapy. These findings support the overall safety of GH treatment when administered and monitored according to current guidelines (2), while reinforcing that the predominant comorbidity burden in TS reflects the underlying chromosomal disorder rather than treatment-related effects.

Approximately 70% of individuals with Turner syndrome experience specific learning difficulties, particularly in mathematics, visuospatial skills, attention, and fine motor function, and behavioral problems such as anxiety, depression, and social difficulties are common during adolescence and adulthood (10). In our adult cohort, educational success showed heterogeneity, ranging from open high school education to doctoral studies, reflecting the variable cognitive and adaptive profile of TS. In the literature, although overall intelligence is usually within the normal range, deficits in executive function, visuospatial processing, social cognition, and emotional regulation may adversely affect academic performance and psychosocial well-being (10). These aspects could not be assessed because of the retrospective nature of the study.

Psychosocial and educational challenges affected nearly one-third of patients, highlighting the importance of early neuropsychological support. Major gaps were noted in transitional care, as none of the adult patients were under regular cardiology follow-up despite known aortic risks. Limitations include the retrospective, single-center design, small sample size, incomplete adult follow-up, and variable documentation. Cardiovascular follow-up was not consistently performed according to TS–specific surveillance protocols, particularly in patients diagnosed earlier in the study period, and some individuals may have undergone non–TS-specific cardiologic evaluations. This heterogeneity, together with the retrospective design, limits the ability to accurately assess cardiovascular comorbidities and to disentangle effects related to growth hormone therapy from those intrinsic to Turner syndrome. Nonetheless, this long-term cohort provides valuable insights into the clinical spectrum and care needs of TS across the lifespan.

Our findings emphasize the critical need for early diagnosis and multidisciplinary care in TS, beginning in childhood and extending into adulthood. Early initiation of GH therapy remains pivotal in maximizing final height, while timely pubertal induction is essential for physical and psychological well-being. Regular and structured screening for cardiovascular, renal, audiological, metabolic, and autoimmune complications is necessary throughout life, including during the transition to adult care. Notably, the absence of cardiology follow-up in all transitioned patients—even those with known cardiac risks—underscores the need for standardized transition protocols and dedicated transition clinics involving pediatric and adult endocrinologists, cardiologists, and other specialists. Furthermore, neurocognitive and psychosocial support services should be integrated early in care plans to optimize educational outcomes and quality of life. Counseling on fertility potential and assisted reproductive options should be offered proactively in adolescence and early adulthood, as evidenced by our patient who achieved successful pregnancy with assisted techniques.

In conclusion, earlier initiation of GH therapy was associated with a trend toward improved final height outcomes in this real-world cohort of patients with TS. Although this association did not reach statistical significance after multivariable adjustment, the observed pattern supports the hypothesis that GH initiation timing may influence growth potential. Larger prospective studies are needed to confirm these findings. Turner syndrome requires lifelong, multidisciplinary management, with emphasis on early diagnosis, individualized treatment, and systematic screening for comorbidities. Structured transition protocols and coordinated follow-up into adulthood are essential to reduce long-term risks and optimize health outcomes.

## Data Availability

The original contributions presented in the study are included in the article/**Supplementary Material**. Further inquiries can be directed to the corresponding author.

## Glossary

ALT: Alanine aminotransferase
BMI: Body mass index
CV: Cardiovascular
DEXA/DXA: Dual-energy X-ray absorptiometry
FH: Final height
GH: Growth hormone
HRT: Hormone replacement therapy
IVF: *In vitro* fertilization
NASH: Nonalcoholic fatty liver disease
OCD: Obsessive–compulsive disorder
SDS: Standard deviation score
T2DM: Type 2 diabetes mellitus
TH: Target height
TS: Turner syndrome
