# Supplementary material for: Turner syndrome across the lifespan: a 25-year single-center experience from neonatal diagnosis to adult outcomes
Source: Front Endocrinol (Lausanne). 2026 Apr 10;17:1771065. doi: 10.3389/fendo.2026.1771065 (PMC13106068; doi:10.3389/fendo.2026.1771065)
Supplement: Supplementary file 1 [file Table1.docx]

**Supplementary Table S1. Multivariable Linear Regression Analysis of Factors Associated with Final Height SDS in GH-treated Turner Syndrome Patients (n = 23)**

**Dependent variable:** Final height SDS

| **Predictor** | **Unstandardized β (B)** | **Standard Error (SE)** | **Standardized β** | **95% CI for B** | **p value** | **VIF** |
| --- | --- | --- | --- | --- | --- | --- |
| **Intercept** | −0.88 | 1.12 | — | −3.24 to 1.48 | 0.45 | — |
| **Age at GH initiation (years)** | −0.16 | 0.10 | −0.31 | −0.37 to 0.05 | 0.12 | 1.38 |
| **GH treatment duration (years)** | −0.11 | 0.14 | −0.19 | −0.33 to 0.10 | 0.27 | 1.42 |
| **Baseline height SDS** | +0.34 | 0.19 | +0.38 | −0.04 to 0.72 | 0.074 | 1.09 |

*Linear regression analysis was performed using the enter method. Variables included were selected a priori based on clinical relevance. Collinearity diagnostics showed acceptable variance inflation factors (VIF < 2 for all variables).*
